# The Maryland State Dental Association

**Published:** 1883-11

**Authors:** 


Maryland State Dental Association.—At a meeting of the dentists of Baltimore and the State of Maryland, held Thursday evening, October 18th, 1883, at the Infirmary of the University of Maryland Dental Department for the purpose of organizing a State Dental Association, a complete organization was effected of the Maryland State Dental Association. Dr. F. J. S. Gorgas was appointed President *pro tem.*, and Dr. B. M. Hopkinson Secretary *pro tem.* Letters of sympathy with the movement were read from many prominent practitioners throughout the State. A constitution, by-laws and code of ethics were prepared and adopted. The next meeting, to be held in Chemical Hall of the University of Maryland, was appointed to take place on the second Thursday in November. The following gentlemen were elected officers for the ensuing year; President, T. J. Smithers, of Easton, Maryland; First Vice-President, Dr. T. H. Davy, of Baltimore; second Vice President, Dr. D. Genese, of Baltimore; Corresponding Secretary, Dr. W. A. Mills, of Baltimore; Recording Secretary, Dr. B. M. Hopkinson, of Baltimore; Dr. A. C. McCurdy, of Towsontown, Maryland, was elected Treasurer; Executive Committee, Drs. B. M. Wilkinson, J. C. Uhler, R. A. Hungerford, all of Baltimore.

